# (*E*)-*N*-{2-[1-(Benzyl­imino)eth­yl]phen­yl}benzamide

**DOI:** 10.1107/S1600536810007610

**Published:** 2010-03-03

**Authors:** Chao-Hsiang Wang, Yi-Chang Liu, Chia-Her Lin, Bao-Tsan Ko

**Affiliations:** a Department of Chemistry, Chung Yuan Christian University, Chung-Li 32023, Taiwan

## Abstract

In the title compound, C_22_H_20_N_2_O, the molecular conformation is supported by an intra­molecular N—H⋯N hydrogen bond, resulting in an almost planar [mean deviation = 0.048 (2) Å] *S*(6) ring. The dihedral angles between the central benzene ring and the imine- and amide-substituted aromatic rings are 76.6 (2) and 11.7 (2)°, respectively.

## Related literature

For background to the application of β-diketiminate-containing metal complexes in ring-opening polymerization, see: Chamberlain *et al.* (2001 ▶); Chisholm *et al.* (2002 ▶). For related structures, see: Gao *et al.* (2008 ▶); Tsai *et al.* (2009 ▶); Liu *et al.* (2009 ▶).
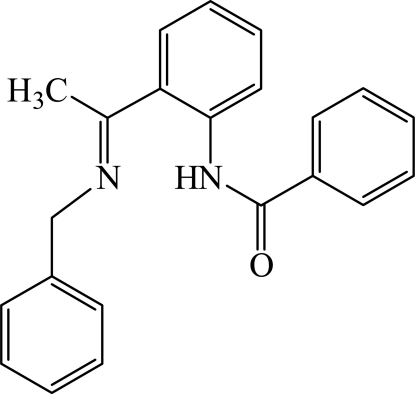


## Experimental

### 

#### Crystal data

C_22_H_20_N_2_O
*M* *_r_* = 328.40
Monoclinic, 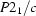

*a* = 10.4213 (4) Å
*b* = 17.0799 (7) Å
*c* = 10.8028 (5) Å
β = 114.394 (2)°
*V* = 1751.18 (13) Å^3^
*Z* = 4
Mo *K*α radiation
μ = 0.08 mm^−1^
*T* = 296 K
0.25 × 0.15 × 0.15 mm

#### Data collection

Bruker APEXII CCD diffractometer
Absorption correction: multi-scan (*SADABS*; Bruker, 2009 ▶) *T*_min_ = 0.985, *T*_max_ = 0.989
20052 measured reflections
4263 independent reflections
2414 reflections with *I* > 2σ(*I*)
*R*_int_ = 0.041

#### Refinement

*R*[*F* ^2^ > 2σ(*F* ^2^)] = 0.053
*wR*(*F* ^2^) = 0.161
*S* = 1.03
4263 reflections
226 parameters
H-atom parameters constrained
Δρ_max_ = 0.20 e Å^−3^
Δρ_min_ = −0.17 e Å^−3^

### 

Data collection: *APEX2* (Bruker, 2009 ▶); cell refinement: *SAINT-Plus* (Bruker, 2009 ▶); data reduction: *SAINT-Plus*; program(s) used to solve structure: *SHELXS97* (Sheldrick, 2008 ▶); program(s) used to refine structure: *SHELXL97* (Sheldrick, 2008 ▶); molecular graphics: *SHELXTL* (Sheldrick, 2008 ▶); software used to prepare material for publication: *SHELXTL*.

## Supplementary Material

Crystal structure: contains datablocks I, global. DOI: 10.1107/S1600536810007610/rk2194sup1.cif

Structure factors: contains datablocks I. DOI: 10.1107/S1600536810007610/rk2194Isup2.hkl

Additional supplementary materials:  crystallographic information; 3D view; checkCIF report

## Figures and Tables

**Table 1:** Hydrogen-bond geometry (Å, °)

*D*—H⋯*A*	*D*—H	H⋯*A*	*D*⋯*A*	*D*—H⋯*A*
N2—H2*B*⋯N1	0.86	1.97	2.670 (2)	138

**Fig. 1.**
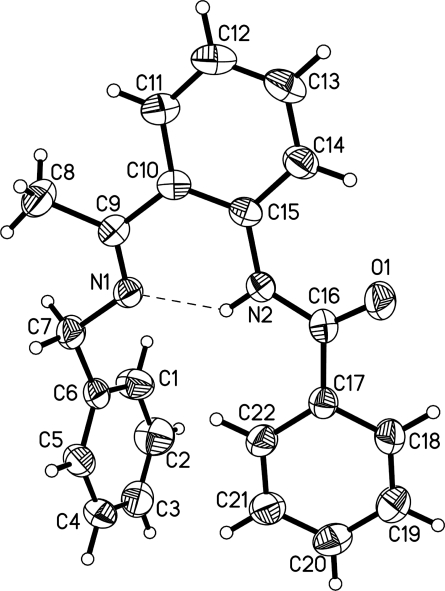
A view of the molecular structure of I with the atom numbering scheme. Displacement ellipsoids are drawn at the 30% probability level. H atoms are presented as a small spheres of arbitrary radius. Intramolecular H-bond drawn by dashed line.

**Fig. 2.**
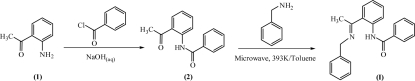
The synthetic procedure of the title compound I.

## supplementary crystallographic information

### Comment

Due to the excellent application of β-diketiminate containing metal complexes in ring-opening polymerization (Chamberlain *et al.*, 2001; Chisholm *et al.*, 2002), structurally-related ligand precursors were synthesized and examined catalytic activities in ring-opening polymerization. For instance, anilido-aldimine (*AA*) ligands have been designed to control the steric or electronic effect to provide a single active metal center for minimizing the side reaction. Recently, a series of *N,N,N*-tridentate *AA* rare-earth metal, magnesium and zinc complexes have demonstrated that the nitrogen atom of pendant arm can coordinate with the metal to increase the sterics and coordination sites of the ligand, creating a single active site nature to initiate the polymerization of ε-caprolactone and *L*-lactide (Gao *et al.*, 2008; Tsai *et al.*, 2009). In order to investigate ligand precursors bearing similar chelating systems and iso-electronic features related to anilido-aldimine ligands, our group is interested in developing new *AA*-like ligands from the aminoacetophenone derivatives. Herein, we report the synthesis and crystal structure of the title compound, (I), a potential *N,N,O*-tridentate *AA*-like ligand for the preparation of aluminum, magnesium and zinc complexes (Scheme 1).

The solid structure of I reveals the phenyl configuration containing one benzamide functionalized group and one benzyl substituted imine group on the *ortho*-position (Fig. 1). It was found that there is an intramolecular N–H···N hydrogen bond between the amide and imine groups. The distance of N···H is substantially shorter than the van der Waals distance of 2.75Å for the N and H atoms. It is interesting to note that the six-member ring (N1/C9/C10/C15/N2/H2B) formed from the N–H···N hydrogen-bond is almost coplanar with the mean deviation of 0.048 (2)Å. These bond distances of benzyl substituted imine group are similar to those found in the crystal structure of (*E*)-*N*-(2-((benzylimino)methyl)phenyl)-2,6-diisopropylaniline (Liu *et al.*, 2009).

### Experimental

The title compound I was synthesized by the following procedures (Fig. 2):

*N*-(2-acetylphenyl)benzamide (2). In a 50 ml round bottom flask, benzoyl chloride (15.5 g, 110.7 mmol) was added to a solution of 2'-aminoacetophenone, (1) (10.0 g, 74.1 mmol) dissolved in 5% NaOH_(aq)_ solution (20 ml). The mixture was stirred vigorously for 2 h and the resultant precipitate was washed with dichloromethane (3×50 ml), followed by deionized water (2×50 ml). The organic layer was dried over anhydrous magnesium sulfate and the solvent was dried under vacuum to give white solids. Yield: 14.09 g (86 %). ^1^H NMR (CDCl_3_, ppm): δ 12.69 (s, 1H, N*H*), 8.97 (d, J = 8.7 Hz, 1H, Ph*H*), 8.06 (d, J = 9.0 Hz, 2H, Ph*H*), 7.95 (d, J = 7.8 Hz, 1H, Ph*H*) , 7.48-7.64 (m, 4H, Ph*H*), 7.15 (t, J = 7.5 Hz, 1H, Ph*H*), 2.70 (s, 3H, *CH*_3_C═*O*).

(*E*)-*N*-(2-(1-(benzylimino)ethyl)phenyl)benzamide I.*N*-(2-acetylphenyl)benzamide, 2 (0.96 g, 4.0 mmol), benzylamine (4.29 g, 40.0 mmol) and activated 4Å molecular sieves (2.0 g) were introduced in a Smith Process Vial^TM^ containing a small stirrer bar. The vial was sealed and heated to 393 K under a microwave reactor for 2 h. Volatile materials were removed under vacuum to give white solids. The desired product was isolated by repeated crystallization from hexane/CH_2_Cl_2_ to give white solids. Colourless crystals were obtained from the saturated *Et*_2_O solution. Yield: 1.10 g (88 %). ^1^H NMR (CDCl_3_, ppm): δ 13.81 (s, 1H, N*H*), 8.82 (d, J = 9.0 Hz, 2H, Ph*H*), 7.64-7.71 (m, 3H, Ph*H*), 7.43 (t, J = 7.2 Hz, 1H, Ph*H*), 7.27-7.32 (m, 6H, Ph*H*), 7.01-7.11 (m, 2H, Ph*H*), 4.75 (s, 2H, PhC*H*_2_), 2.40 (s, 3H, C*H*_3_C═N).

### Refinement

The H atoms were placed in idealized positions and constrained to ride on their parent atoms, with C–H = 0.93Å with *U*_iso_(H) = 1.2 *U*_eq_(C) for phenyl hydrogen; 0.96Å with *U*_iso_(H) = 1.5 *U*_eq_(C) for CH_3_ group; 0.97Å with *U*_iso_(H) = 1.2 *U*_eq_(C) for CH_2 _group; N–H = 0.86Å with *U*_iso_(H) = 1.2 *U*_eq_(N).

### Crystal data

**Table:**

C_22_H_20_N_2_O	*F*(000) = 696
*M**_r_* = 328.40	*D*_x_ = 1.246 Mg m^−^^3^
Monoclinic, *P*2_1_/*c*	Mo *K*α radiation, λ = 0.71073 Å
Hall symbol: -P 2ybc	Cell parameters from 1657 reflections
*a* = 10.4213 (4) Å	θ = 1.7–28.3°
*b* = 17.0799 (7) Å	µ = 0.08 mm^−^^1^
*c* = 10.8028 (5) Å	*T* = 296 K
β = 114.394 (2)°	Block, colourless
*V* = 1751.18 (13) Å^3^	0.25 × 0.15 × 0.15 mm
*Z* = 4	

### Data collection

**Table:**

Bruker APEXII CCD diffractometer	4263 independent reflections
Radiation source: fine-focus sealed tube	2414 reflections with *I* > 2σ(*I*)
graphite	*R*_int_ = 0.041
Detector resolution: 8.3333 pixels mm^-1^	θ_max_ = 28.3°, θ_min_ = 2.4°
φ and ω scans	*h* = −13→13
Absorption correction: multi-scan (*SADABS*; Bruker, 2009)	*k* = −22→22
*T*_min_ = 0.985, *T*_max_ = 0.989	*l* = −14→11
20052 measured reflections	

### Refinement

**Table:**

Refinement on *F*^2^	Primary atom site location: structure-invariant direct methods
Least-squares matrix: full	Secondary atom site location: difference Fourier map
*R*[*F*^2^ > 2σ(*F*^2^)] = 0.053	Hydrogen site location: inferred from neighbouring sites
*wR*(*F*^2^) = 0.161	H-atom parameters constrained
*S* = 1.03	*w* = 1/[σ^2^(*F*_o_^2^) + (0.071*P*)^2^ + 0.1853*P*] where *P* = (*F*_o_^2^ + 2*F*_c_^2^)/3
4263 reflections	(Δ/σ)_max_ < 0.001
226 parameters	Δρ_max_ = 0.20 e Å^−^^3^
0 restraints	Δρ_min_ = −0.17 e Å^−^^3^

### Special details

**Table:**

Geometry. All s.u.'s (except the s.u. in the dihedral angle between two l.s. planes) are estimated using the full covariance matrix. The cell s.u.'s are taken into account individually in the estimation of s.u.'s in distances, angles and torsion angles; correlations between s.u.'s in cell parameters are only used when they are defined by crystal symmetry. An approximate (isotropic) treatment of cell s.u.'s is used for estimating s.u.'s involving l.s. planes.
Refinement. Refinement of *F*^2^ against ALL reflections. The weighted *R*-factor wR and goodness of fit *S* are based on *F*^2^, conventional *R*-factors *R* are based on *F*, with *F* set to zero for negative *F*^2^. The threshold expression of *F*^2^ > σ(*F*^2^) is used only for calculating *R*-factors(gt) etc. and is not relevant to the choice of reflections for refinement. *R*-factors based on *F*^2^ are statistically about twice as large as those based on *F*, and *R*-factors based on ALL\ data will be even larger.

### Fractional atomic coordinates and isotropic or equivalent isotropic displacement parameters (Å^2^)

**Table:**

	*x*	*y*	*z*	*U*_iso_*/*U*_eq_	
O1	0.81960 (13)	0.10603 (8)	0.23208 (15)	0.0812 (4)	
N1	0.34187 (14)	0.01877 (8)	0.15594 (15)	0.0574 (4)	
N2	0.60695 (13)	0.06148 (8)	0.21381 (14)	0.0539 (4)	
H2B	0.5180	0.0649	0.1640	0.065*	
C1	0.1425 (2)	0.16265 (13)	0.0401 (2)	0.0861 (6)	
H1A	0.1731	0.1658	0.1339	0.103*	
C2	0.0954 (3)	0.22894 (14)	−0.0374 (3)	0.1020 (8)	
H2A	0.0960	0.2765	0.0047	0.122*	
C3	0.0482 (2)	0.22600 (14)	−0.1736 (3)	0.0894 (7)	
H3A	0.0147	0.2710	−0.2255	0.107*	
C4	0.0501 (2)	0.15672 (16)	−0.2343 (2)	0.0881 (7)	
H4A	0.0183	0.1543	−0.3283	0.106*	
C5	0.09905 (19)	0.08970 (12)	−0.1574 (2)	0.0710 (5)	
H5A	0.1006	0.0427	−0.2003	0.085*	
C6	0.14522 (16)	0.09148 (10)	−0.01899 (19)	0.0572 (4)	
C7	0.19123 (18)	0.01887 (11)	0.0651 (2)	0.0694 (5)	
H7A	0.1371	0.0135	0.1191	0.083*	
H7B	0.1708	−0.0261	0.0051	0.083*	
C8	0.2975 (2)	−0.09913 (11)	0.2593 (2)	0.0810 (6)	
H8A	0.2029	−0.0924	0.1920	0.122*	
H8B	0.2981	−0.0969	0.3484	0.122*	
H8C	0.3330	−0.1490	0.2468	0.122*	
C9	0.38926 (17)	−0.03509 (9)	0.24489 (18)	0.0539 (4)	
C10	0.54103 (17)	−0.03557 (9)	0.34125 (17)	0.0528 (4)	
C11	0.5834 (2)	−0.08470 (11)	0.4543 (2)	0.0687 (5)	
H11A	0.5159	−0.1155	0.4664	0.082*	
C12	0.7205 (2)	−0.08954 (13)	0.5482 (2)	0.0807 (6)	
H12A	0.7449	−0.1231	0.6222	0.097*	
C13	0.8209 (2)	−0.04467 (14)	0.5322 (2)	0.0804 (6)	
H13A	0.9141	−0.0478	0.5954	0.096*	
C14	0.78470 (19)	0.00524 (11)	0.4231 (2)	0.0684 (5)	
H14A	0.8540	0.0359	0.4139	0.082*	
C15	0.64589 (17)	0.01073 (9)	0.32591 (17)	0.0519 (4)	
C16	0.69092 (17)	0.10573 (9)	0.17378 (18)	0.0551 (4)	
C17	0.61755 (17)	0.15684 (9)	0.05235 (18)	0.0524 (4)	
C18	0.6931 (2)	0.21764 (12)	0.0312 (2)	0.0797 (6)	
H18A	0.7863	0.2253	0.0921	0.096*	
C19	0.6337 (3)	0.26723 (13)	−0.0778 (3)	0.0901 (7)	
H19A	0.6864	0.3082	−0.0893	0.108*	
C20	0.4985 (2)	0.25675 (12)	−0.1688 (2)	0.0795 (6)	
H20A	0.4584	0.2902	−0.2428	0.095*	
C21	0.4218 (2)	0.19642 (14)	−0.1506 (2)	0.0801 (6)	
H21A	0.3294	0.1886	−0.2132	0.096*	
C22	0.48016 (18)	0.14713 (11)	−0.0403 (2)	0.0674 (5)	
H22A	0.4262	0.1069	−0.0284	0.081*	

### Atomic displacement parameters (Å^2^)

**Table:**

	*U*^11^	*U*^22^	*U*^33^	*U*^12^	*U*^13^	*U*^23^
O1	0.0496 (7)	0.0893 (9)	0.0919 (11)	−0.0078 (6)	0.0164 (7)	0.0193 (8)
N1	0.0473 (7)	0.0614 (8)	0.0613 (10)	0.0001 (6)	0.0202 (7)	0.0087 (7)
N2	0.0451 (7)	0.0590 (8)	0.0526 (9)	−0.0016 (6)	0.0151 (6)	0.0037 (7)
C1	0.1098 (18)	0.0804 (14)	0.0623 (14)	0.0046 (12)	0.0295 (13)	−0.0019 (11)
C2	0.124 (2)	0.0714 (14)	0.107 (2)	0.0156 (13)	0.0447 (17)	0.0016 (14)
C3	0.0634 (12)	0.0809 (15)	0.103 (2)	0.0037 (10)	0.0141 (13)	0.0290 (14)
C4	0.0692 (13)	0.1176 (19)	0.0583 (14)	−0.0153 (12)	0.0071 (10)	0.0158 (14)
C5	0.0644 (11)	0.0801 (13)	0.0617 (14)	−0.0058 (9)	0.0193 (10)	−0.0063 (10)
C6	0.0400 (8)	0.0653 (10)	0.0603 (12)	−0.0041 (7)	0.0146 (8)	0.0018 (8)
C7	0.0484 (10)	0.0723 (11)	0.0785 (14)	−0.0061 (8)	0.0172 (9)	0.0115 (10)
C8	0.0718 (12)	0.0674 (11)	0.1066 (18)	0.0008 (9)	0.0396 (12)	0.0227 (12)
C9	0.0561 (10)	0.0506 (9)	0.0610 (11)	0.0043 (7)	0.0304 (9)	0.0017 (8)
C10	0.0577 (10)	0.0524 (9)	0.0511 (11)	0.0099 (7)	0.0252 (8)	0.0021 (8)
C11	0.0750 (12)	0.0710 (12)	0.0636 (13)	0.0120 (9)	0.0322 (11)	0.0137 (10)
C12	0.0889 (15)	0.0903 (14)	0.0606 (14)	0.0246 (12)	0.0286 (12)	0.0219 (11)
C13	0.0656 (12)	0.1040 (16)	0.0579 (13)	0.0167 (11)	0.0117 (10)	0.0066 (12)
C14	0.0567 (11)	0.0828 (12)	0.0584 (12)	0.0033 (9)	0.0165 (9)	0.0035 (10)
C15	0.0533 (9)	0.0550 (9)	0.0460 (10)	0.0076 (7)	0.0191 (8)	−0.0013 (7)
C16	0.0496 (9)	0.0519 (9)	0.0618 (11)	−0.0047 (7)	0.0211 (8)	−0.0059 (8)
C17	0.0537 (9)	0.0488 (8)	0.0563 (11)	−0.0027 (7)	0.0244 (8)	−0.0051 (8)
C18	0.0730 (13)	0.0726 (12)	0.0773 (15)	−0.0218 (10)	0.0149 (11)	0.0089 (11)
C19	0.0976 (17)	0.0733 (13)	0.0908 (17)	−0.0205 (12)	0.0303 (14)	0.0143 (12)
C20	0.0893 (15)	0.0771 (13)	0.0766 (15)	0.0122 (11)	0.0388 (13)	0.0208 (11)
C21	0.0588 (11)	0.1083 (16)	0.0723 (15)	0.0068 (11)	0.0261 (11)	0.0211 (12)
C22	0.0554 (10)	0.0817 (12)	0.0670 (13)	−0.0036 (9)	0.0273 (10)	0.0124 (10)

### Geometric parameters (Å, °)

**Table:**

O1—C16	1.2237 (19)	C9—C10	1.492 (2)
N1—C9	1.273 (2)	C10—C11	1.394 (2)
N1—C7	1.468 (2)	C10—C15	1.413 (2)
N2—C16	1.355 (2)	C11—C12	1.371 (3)
N2—C15	1.406 (2)	C11—H11A	0.9300
N2—H2B	0.8600	C12—C13	1.364 (3)
C1—C2	1.373 (3)	C12—H12A	0.9300
C1—C6	1.379 (3)	C13—C14	1.375 (3)
C1—H1A	0.9300	C13—H13A	0.9300
C2—C3	1.346 (3)	C14—C15	1.396 (2)
C2—H2A	0.9300	C14—H14A	0.9300
C3—C4	1.357 (3)	C16—C17	1.496 (2)
C3—H3A	0.9300	C17—C22	1.377 (2)
C4—C5	1.382 (3)	C17—C18	1.378 (2)
C4—H4A	0.9300	C18—C19	1.373 (3)
C5—C6	1.369 (3)	C18—H18A	0.9300
C5—H5A	0.9300	C19—C20	1.356 (3)
C6—C7	1.494 (2)	C19—H19A	0.9300
C7—H7A	0.9700	C20—C21	1.367 (3)
C7—H7B	0.9700	C20—H20A	0.9300
C8—C9	1.503 (2)	C21—C22	1.378 (3)
C8—H8A	0.9600	C21—H21A	0.9300
C8—H8B	0.9600	C22—H22A	0.9300
C8—H8C	0.9600		
			
C9—N1—C7	118.67 (14)	C11—C10—C9	118.39 (15)
C16—N2—C15	128.61 (14)	C15—C10—C9	124.30 (15)
C16—N2—H2B	115.7	C12—C11—C10	122.73 (18)
C15—N2—H2B	115.7	C12—C11—H11A	118.6
C2—C1—C6	121.0 (2)	C10—C11—H11A	118.6
C2—C1—H1A	119.5	C13—C12—C11	119.44 (19)
C6—C1—H1A	119.5	C13—C12—H12A	120.3
C3—C2—C1	120.9 (2)	C11—C12—H12A	120.3
C3—C2—H2A	119.6	C12—C13—C14	120.24 (19)
C1—C2—H2A	119.6	C12—C13—H13A	119.9
C2—C3—C4	119.3 (2)	C14—C13—H13A	119.9
C2—C3—H3A	120.3	C13—C14—C15	121.23 (18)
C4—C3—H3A	120.3	C13—C14—H14A	119.4
C3—C4—C5	120.4 (2)	C15—C14—H14A	119.4
C3—C4—H4A	119.8	C14—C15—N2	122.02 (15)
C5—C4—H4A	119.8	C14—C15—C10	119.03 (16)
C6—C5—C4	121.0 (2)	N2—C15—C10	118.95 (14)
C6—C5—H5A	119.5	O1—C16—N2	123.57 (16)
C4—C5—H5A	119.5	O1—C16—C17	120.22 (15)
C5—C6—C1	117.39 (18)	N2—C16—C17	116.21 (14)
C5—C6—C7	121.67 (18)	C22—C17—C18	117.66 (17)
C1—C6—C7	120.88 (19)	C22—C17—C16	124.64 (15)
N1—C7—C6	113.17 (14)	C18—C17—C16	117.69 (16)
N1—C7—H7A	108.9	C19—C18—C17	121.37 (19)
C6—C7—H7A	108.9	C19—C18—H18A	119.3
N1—C7—H7B	108.9	C17—C18—H18A	119.3
C6—C7—H7B	108.9	C20—C19—C18	120.40 (19)
H7A—C7—H7B	107.8	C20—C19—H19A	119.8
C9—C8—H8A	109.5	C18—C19—H19A	119.8
C9—C8—H8B	109.5	C19—C20—C21	119.3 (2)
H8A—C8—H8B	109.5	C19—C20—H20A	120.4
C9—C8—H8C	109.5	C21—C20—H20A	120.4
H8A—C8—H8C	109.5	C20—C21—C22	120.6 (2)
H8B—C8—H8C	109.5	C20—C21—H21A	119.7
N1—C9—C10	119.97 (14)	C22—C21—H21A	119.7
N1—C9—C8	122.63 (16)	C17—C22—C21	120.66 (17)
C10—C9—C8	117.40 (15)	C17—C22—H22A	119.7
C11—C10—C15	117.31 (16)	C21—C22—H22A	119.7
			
C6—C1—C2—C3	1.1 (4)	C13—C14—C15—N2	179.93 (17)
C1—C2—C3—C4	−1.3 (4)	C13—C14—C15—C10	0.5 (3)
C2—C3—C4—C5	0.4 (3)	C16—N2—C15—C14	6.6 (3)
C3—C4—C5—C6	0.6 (3)	C16—N2—C15—C10	−174.05 (15)
C4—C5—C6—C1	−0.8 (3)	C11—C10—C15—C14	−0.1 (2)
C4—C5—C6—C7	176.70 (17)	C9—C10—C15—C14	179.92 (15)
C2—C1—C6—C5	−0.1 (3)	C11—C10—C15—N2	−179.47 (14)
C2—C1—C6—C7	−177.6 (2)	C9—C10—C15—N2	0.5 (2)
C9—N1—C7—C6	170.89 (16)	C15—N2—C16—O1	1.6 (3)
C5—C6—C7—N1	114.16 (19)	C15—N2—C16—C17	−177.54 (14)
C1—C6—C7—N1	−68.4 (2)	O1—C16—C17—C22	162.53 (18)
C7—N1—C9—C10	−178.33 (15)	N2—C16—C17—C22	−18.3 (2)
C7—N1—C9—C8	0.8 (3)	O1—C16—C17—C18	−17.0 (3)
N1—C9—C10—C11	166.75 (16)	N2—C16—C17—C18	162.18 (17)
C8—C9—C10—C11	−12.5 (2)	C22—C17—C18—C19	0.4 (3)
N1—C9—C10—C15	−13.2 (2)	C16—C17—C18—C19	179.96 (19)
C8—C9—C10—C15	167.56 (16)	C17—C18—C19—C20	−0.8 (4)
C15—C10—C11—C12	−0.3 (3)	C18—C19—C20—C21	0.2 (4)
C9—C10—C11—C12	179.70 (17)	C19—C20—C21—C22	0.7 (3)
C10—C11—C12—C13	0.2 (3)	C18—C17—C22—C21	0.6 (3)
C11—C12—C13—C14	0.3 (3)	C16—C17—C22—C21	−178.98 (17)
C12—C13—C14—C15	−0.7 (3)	C20—C21—C22—C17	−1.1 (3)

### Hydrogen-bond geometry (Å, °)

**Table:**

*D*—H···*A*	*D*—H	H···*A*	*D*···*A*	*D*—H···*A*
N2—H2B···N1	0.86	1.97	2.670 (2)	138
